# Development of a supportive care needs eHealth application for patients with cervical cancer undergoing surgery: a feasibility study

**DOI:** 10.1186/s12913-023-10437-3

**Published:** 2024-01-02

**Authors:** Yongxia Song, Lili Xia, Xiaodi Ju, Wenjing Wang, Xiaoling Ge, Jingfang Hong

**Affiliations:** 1 School of Nursing, Anhui Medical University, No. 81 Mei Shan Road, Shu Shan District, Hefei City, Anhui Province China; 2 The First Affiliated Hospital of Anhui Medical University, No, 218 Ji Xi Road, Shu Shan District, Hefei City, Anhui Province China; 3 The First Affiliated Hospital of University of Science and Technology of China, No. 17 Lu Jiang Road, Lu Yang District, Hefei City, Anhui Province China; 4 Nursing International Collaboration Research Center of Anhui Province, Hefei, China

**Keywords:** Cervical cancer, Supportive care needs, Supply, Application, eHealth, Feasibility study

## Abstract

**Purpose:**

To inform the development of an eHealth application for patients with cervical cancer for monitoring supportive care needs, perceived care supply and quality of life.

**Methods:**

A mixed-method design was used. The 19-month process involved five phases: (1) a literature review to screen the components of applications, (2) a cross-sectional needs assessment for patients with cervical cancer to define the needs and application program frame, (3) expert consultation to refine the draft, (4) software development, and (5) pilot testing and user comment collection. Patients in the intervention group received a 7-day application intervention combined with usual care. Supportive care needs, perceived care supply, quality of life and user’s additional comments were collected.

**Results:**

The literature review results in phase 1 revealed the importance of full preparation, especially a supportive care needs assessment, before application development. Subsequent supportive care needs investigation in phase 2 revealed that the most urgent needs were informational needs and privacy protection. In phase 3, 43 expert recommendations for application improvement were refined. The new application contained the patient and the health care professional portal in phase 4. Then, on Day 7, there existed score changes of the outcome measures in both intervention and control group. Users had a positive experience with the application.

**Conclusions:**

This study demonstrates the feasibility of applications targeting access to supportive care, which may be effective for improving the outcome measures but needed to be evaluated in future studies.

**Supplementary Information:**

The online version contains supplementary material available at 10.1186/s12913-023-10437-3.

## Introduction

Cervical cancer is one of the most common malignant tumors that threatens women’s health worldwide. Surgery remains one of the main treatment methods [1]. Patients with cervical cancer have to face the specific adverse effects of the cancer and surgical treatment that are not found in other cancers, which might lead to a negative impact on supportive care needs [2]. In addition, supportive care needs for patients with cervical cancer vary depending on different treatment stages [3], such as information about the cancer diagnosis or treatment stage, specific confront postoperative dysuria after surgery and sexual dysfunction needs during survivorship [4].

Health care professionals have the responsibility to promote matching between patients’ supportive care needs and supportive care services for their medical specialty [5]. Due to the lack of awareness of the active utilization of supportive care services, various unmet supportive care needs of patients with cervical cancer have been exhibited [6]. A study showed that 88% of long-term cancer survivors (N = 2,107) preferred oncologists to be involved in their cancer follow-up care, but only 60% reported an oncologist visit after treatment [7]. Kuroki et al.’s investigation of needs among women with abnormal cervical cancer screening results also found that 59% (N = 100) of women had one or more unmet basic needs that urgently required professional needs management guidance from health care professionals [8]. An awareness of the above problem might facilitate early identification of women’s unmet needs and enable individualized follow-up care adjusted for these needs [7].

Interventions aimed at monitoring supportive care needs should be available. However, how to help women undergoing cervical cancer treatment to manage their supportive care needs throughout the hospital stay and while residing at home has been less explored [4]. Evidence for supportive care needs management strategies provided by health care professionals for women has mainly focused on the stage of cervical cancer screening, cancer prevention systems [9], informal social support systems and follow-up monitoring for postoperative patients with early-stage disease [10], and symptom management during oncological treatment [11]. Tailoring of interventions to cancer patients’ needs has been argued to be a prerequisite for their successful rehabilitation [12]. Virtually, the needs management intervention approaches have mainly focused on face-to-face counseling, telephone follow-up, web-based intervention, or the combination of the three and are typically offered by health care professionals [6, 13]. Such approaches, although promising, have encountered problems in effectively motivating individual self-management and difficulties in functioning as a navigation instrument for personalized supportive care [14].

In recent years, mobile applications have been gradually applied to manage supportive care needs in patients with cancer due to their practical, accessible, real-time and social-distancing support advantages. The China mobile consumer survey report released by Deloitte Consulting found that mobile phone ownership in China was as high as 96%, exceeding the global average of 6% in 2018 [15]. In addition, the frequency of mobile phone use was very high, with over 90% of users using such phones every day [15]. Indeed, one in five mobile phone users had already downloaded at least one health-related application [16]. The effects of mobile health promotion intervention have skyrocketed with demonstrated efficacy and are considered a solution to meet patients’ individual supportive care needs [14]. For example, the application “Oncokompas”, designed to provide personalized information through a physical and psychological assessment of patients with cancer, has shown effectiveness in continuously improving quality of life [17]. The rapid penetration and demonstrated efficacy of mobile phones suggested the feasibility of using mobile technologies to manage supportive care needs among patients with cancer. However, the effect of supportive care needs management using a mobile phone program for patients with cervical cancer undergoing surgery warrants further study. Therefore, this study was aimed to develop a mobile application and inform the targeted management of supportive care needs for patients with cervical cancer undergoing surgery, thus improving personalized access to supportive care.

## Methods

**Fig. 1:**
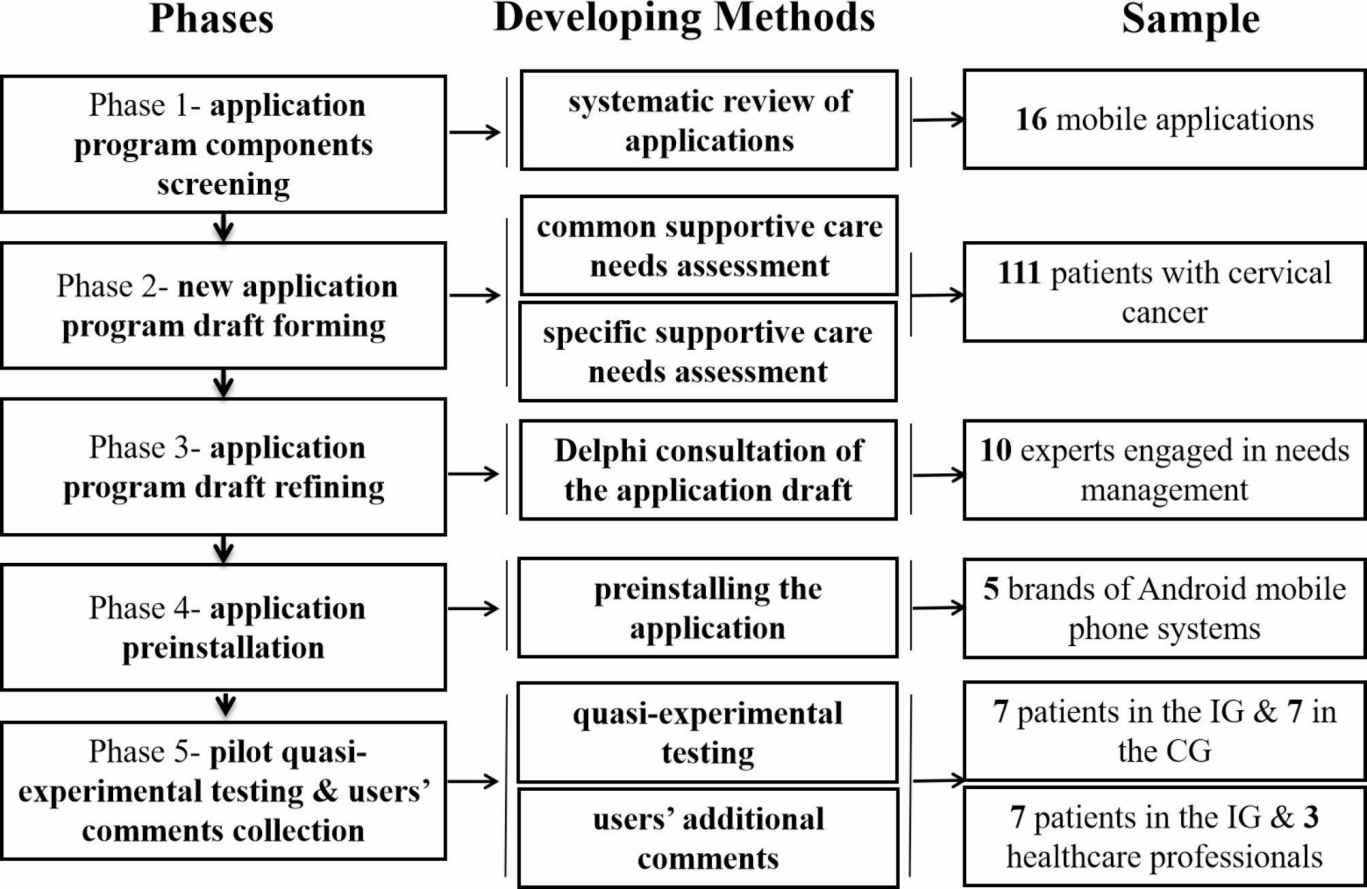
Flow diagram of the study. Note: IG: intervention group; CG: control group. Sample means the corresponding subjects in each phase

According to definition and aiming of a feasibility study concluded by Eldridge et al. [18], an overarching concept for studies assessing whether a future study, project or development could be done and answered the question “can we do this?”, we reinspected the process of a feasibility study that required. We examined the following features of a feasibility needed, such as numbers of eligible patients; characteristics of the proposed primary outcome measure; availability of required data; and acceptability, appropriateness, fidelity, and coverage of the implementation science [19]. Then a five-phase method was summed up to clarify the application program developing process of our feasibility study and anticipate to give the answer “yes, we can!”. The study was carried out between September 15, 2019, and March 20, 2022, and was part of a series of supportive cancer care studies. In phase 1, we screened the possible components of a mobile application for supportive care needs management using a systematic literature review. In phase 2, we defined the existing common and specific supportive care needs of patients with cervical cancer undergoing surgery to construct the frame of the application and the prior needs. In phase 3, we held expert consultations to revise the structure and content of the prototype mobile application program. In phase 4, software validation and testing of the algorithm was developed to test the compatibility and stability in the Android system. In phase 5, we host a pilot quasi-experimental study to evaluate the primary usability of the new development application. The flow diagram and each phase are shown in Fig. 1.

### Phase 1: application program components screening

We conducted a literature review concerning the development and usability evaluation approaches for mobile health applications in supportive care interventions for patients with cancer [20]. The eligibility criteria of the original studies are shown in supplementary materials (1) A methodological quality evaluation was conducted using the standardized QualSyst evaluation tool, which included a qualitative scale and a quantitative scale scoring systems [21]. The qualitative scale included 10 items with scores from 0 to (2) The total score ranged from 0 to 20. A summary score was calculated for each study by summing the obtained scores across the 10 items and dividing them by the total possible score of 20. The quantitative scale included 14 items with scores from 0 to 2, and the possibility of scoring ‘not applicable’ (‘not applicable’ items was excluded from the calculation of the summary score). The total score ranged from 0 to 28. The summary score was calculated by summing the total obtained scores across the relevant items and dividing that by the total possible score (i.e., 28 minus (the number of ‘not applicable’ items multiplied by 2)).

### Phase 2: New application program draft formation

Patients’ supportive care needs were assessed by common and specific parts of the needs assessment scales. The common portion was a 49-item Professional Nursing Support Scale (PNSS), which was used to test the common supportive care needs and perceived support supply for patients with cancer from nurses [22]. It contained four domains: informational needs, technical needs, psychological/emotional needs and care coordination and communication needs. The specific portion was a 25-item specific professional nursing support scale- gynecological cancer (PNSS-GC), which was used to evaluate the specific professional supportive care needs and supply for patients with gynecological cancer [23]. The two scales were rated for the “needs” and “supply” subscales, which provided responses on a five-point Likert scale (ranging from 1 = no need/supply to 5 = always need/supply). Higher total scores indicated more supportive care needs and more perceived supply. For quantitative investigation, a convenience sampling method was used. Cervical cancer inpatients’ eligibility criteria are shown in supplementary materials 1.

### Phase 3: application program draft refinement

Expert consultation focused on issues regarding whether the structure and content of the application program was readable and suitable for patients with cervical cancer undergoing surgery. The inclusion criteria for experts were as follows: (a) majored in medicine or nursing and practiced gynecology and obstetrics, (b) had over five years of work experience in oncology or gynecology and obstetrics, and (c) had a bachelor’s degree or higher. A standardized semistructured scale with unified guidance language and a five-point rating scale (1 = very not important, 5 = very important) regarding the application draft was distributed to the experts via e-mail. Expert reviews were described to validate the requirements of the program draft (version 1.0). The experts were instructed to read the draft in detail and needed to write both positive and negative comments as tracked changes in the text of the draft. The comments were returned to the first author via e-mail. The feedback of the expert consultation enriched the prototype of the application.

### Phase 4: application compatibility testing

For various Android device models and operating system versions on the market, Android-based developers were needed to carefully test the compatibility of their developing application to ensure the user experience. One possible way to test the compatibility of Android applications was to leverage general testing tools to generate tests on one device and replay the tests on the other devices to expose incompatible application behaviors [24]. We completed the human-computer interaction, application data manipulation logic and interface to match the prototype mobile application supported by the software development technical team. Then, we tested the loopholes and disadvantages of the iterative testing system of the mobile application. Five different brands (Oppo, Huawei, Xiaomi, Vivo and Samsung) of Android mobile systems were selected and preinstalled. The phenomena of whether the system ran slowly and flashed back when the system had too much cache was monitored to judge the compatibility and stability of the application. Measures of guiding the user to clean the cache, closing possible conflicting applications, or restarting the application were taken to minimize the unstable phenomena. The frequency of flashbacks of the mobile application was collected, and flashbacks occurring fewer than three times per day per user were allowed.

### Phase 5: pilot quasi-experimental testing & user comments collection

This phase was a pilot quasi-experimental design. Data collection occurred within an inpatient gynecological oncology department in an affiliated hospital of a medical university. Participants in the two groups completed the PNSS, PNSS-GC and quality of life (QoL) scale (Functional Assessment of Cancer Therapy-Cervix subscale, FACT-Cx) at baseline and one week later. The FACT-Cx was the Functional Assessment of Cancer Therapy-General scale (27 items) plus the Cervix subscale (15 additional items) [25]. A five-point scale from 0 to 4 was applied to the FACT-Cx, where 0 was ‘not at all’ and 4 was ‘very much’. The range of possible scores was 0 to 168. Patients were asked to score how they felt that day and during the previous 7 days, with a higher score indicating better QoL.

**Fig. 2:**
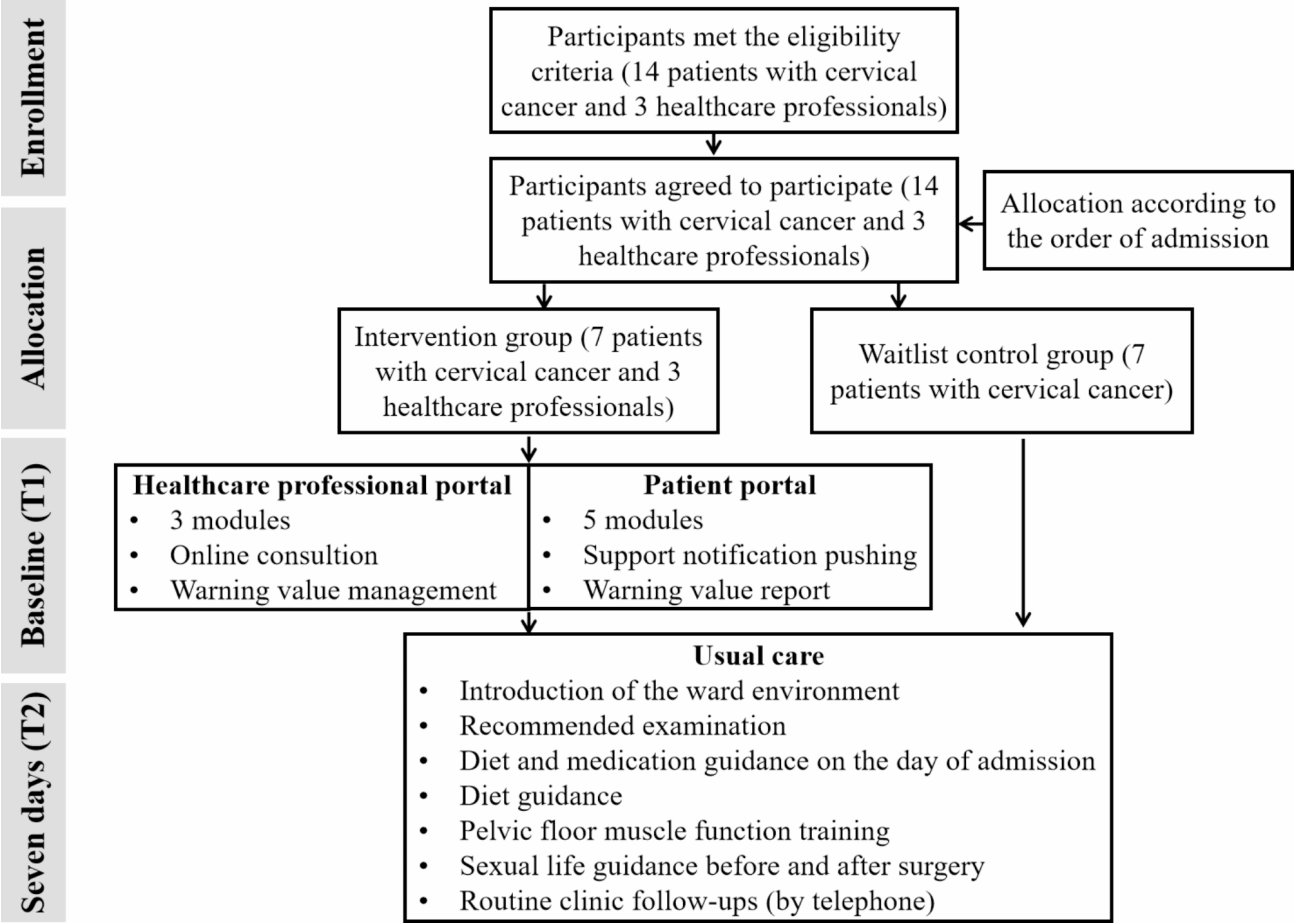
The pilot quasi-experimental testing flowchart

Participants were grouped according to the order of admission. Within the control group (CG), there would be no additional intervention, and care would continue as normally received from health care professionals (see Fig. 2). There were no restrictions related to accessing the internet or participating in other self-help activities. Participants in the CG would install the application after the pilot study. Participants allocated to the intervention group (IG) were given access to a mobile-based 7-day supportive care application on the day of admission: named the Womb Guard (WG). The mobile application named Womb Guard refers to health assistance of the female womb. In the IG, we recruited users, including both patients with cervical cancer undergoing surgery and health care professionals, by using a purposive sampling method considering the differences in age, gender, education level, and mobile phone systems. The eligibility criteria of the participants are also shown in supplementary materials 1. The health care professionals were nurses and physicians who had provided supportive care for patients during hospitalization and follow-ups by using the WG web-based health care professional portal. Real-time online consultation feedback was provided by health care professionals through the interaction module. The page views on the patient portal and records of the platform interactions were captured. The users’ (patients and health care managers) additional comments were also collected after the intervention.

**Fig. 3:**
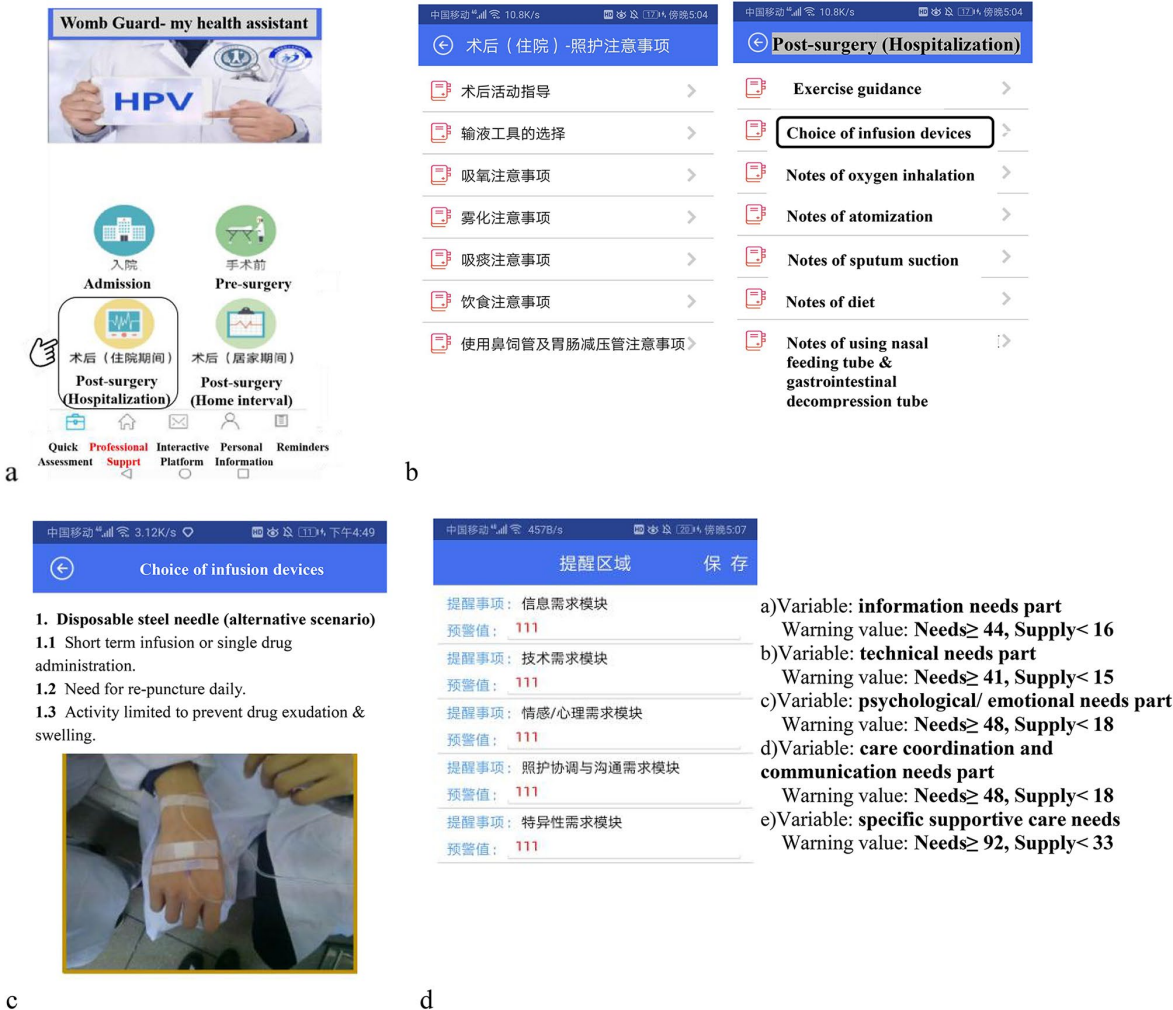
Parts of the interface of Womb Guard. Note: when users entered the interface of Womb Guard and click the menu of the “Post-surgery (Hospitalization)” (Fig. 3-a), a list of post-operative announcements will be presented (Fig. 3-b, the left was the original interface in Chinese, and the right was translated in English). Figure 3-c shows the part of the alternative scenario when users press the “Choice of infusion devices” button. Figure 3-d shows division method of needs and supply to set a warning value and the interface of the reminder area. The red number “111” was just a pseudo-value entering randomly during system iteration. Words on the right also shows the warning value of each domain or the total score of the PNSS and PNSS-GC

Information on the WG patient-portal was presented in modules using texts or videos based on the above application development process. In addition, a push notification reminded participants to participate in the modules and stated the date of the next follow-up. Upon logging into the WG, the participants completed a baseline quick needs assessment (both the “needs” and the “supply” part) and QoL evaluation. The web-based portal then fetched the warning values and pushed targeted supportive notifications according to the results of the needs assessment. The possible higher needs and lower supply were screened as a warning value, which would be set as evidence for the interactive platform (see Fig. 3-d). The warning value setting was based on 27% of the domain or the total score on the PNSS and PNSS-GC. Higher needs meant 73% or greater of the domain or the total score, and lower supply meant less than 27% of the score. For example, the informational needs domain of the PNSS contained 12 items (with a score ranging from 12 to 60). Once the higher needs score was 44 or above and the lower supply was less than 16 according to the Quick Assessment result, a warning value push notification would appear in the patient’s Reminder module, which would be evidence for an interactive platform and push notification.

In the IG, users’ additional comments were collected seven days later by telephone after they used the application. Additional questions were asked as follows: Is the content of the application understandable? If not, what are the main aspects of concern? In which ways does the application benefit you? What problems did you encountered during the application use, and how did you solve them? Would you like to continue using the application in the future and why? What are your suggestions about improving the application?

### Data analysis

Quantitative data, such as the scores of needs, were saved and analyzed using IBM software, SPSS version 24.0. Categorized variables, such as gender, were presented with descriptive statistics, such as percentage and frequency. Data were examined to evaluate for normal distribution. Continuous variables are presented herein as the mean and standard deviation (SD) or as the median and interquartile range (IQR). As for considering of our small sample size analysis might reveal the lack of power, that carried the risk of both false negative results and unreliable positive results [19], we did not calculate the power of *p*-values but show the changes of the score in groups in our pilot study phase. User comments were narrated and summarized according to related questions to identify the major themes and key exemplary quotes.

## Results

### Phase 1: application program components screening

The needs management of treatment-stage patients with cervical cancer has been poorly studied. In this phase, we systematically searched and reviewed the development methods of mobile phone-based application interventions for patients with no restrictions on cancer types to screen the application components of needs management suited for those with cervical cancer. In total, 13 articles with 16 applications were included for systematic analysis. The PRISMA flow diagram of the literature review is shown in Supplementary Information 2. Important information about the search strategy, the included articles list, the detailed data and the quality evaluation is shown in Supplementary Information 3. Information about the cancer type, primary outcomes, name of the application, module of the application, and application development method was extracted. The QualSyst score of these included studies ranged from 0.80 to 1.00. In those 16 applications, there was no such application that acted as a navigation instrument to personalize supportive cancer care needs and supply for cervical cancer patients undergoing surgery.

### Phase 2: New application program draft formation

The results of phase 2 were a secondary analysis from our previous validation study of the specific PNSS-GC [23]. In that validation study, a cross-sectional design was used to identify the common and specific nursing supportive care needs of patients with gynecological cancer by using the PNSS and PNSS-GC scales. A total of 280 gynecological cancer patients were enrolled through a convenience sampling method. Supplementary materials 1 shows the patients’ eligibility criteria in this phase. Patients were recruited from four departments of three affiliated hospitals with a university from August to November of 2018. All recruited patients signed an informed consent form. Voluntary participation, anonymity, and confidentiality were ensured throughout the investigation.

Data from 111 patients with cervical cancer undergoing surgery were used for analysis in phase 2 of this feasibility study to define the common and specific supportive care needs by using the “needs” part of the PNSS and PNSS-GC. These participants were 23 to 67 years of age (mean: 47.94; standard deviation, SD: 8.10). Among the 111 patients, 99 (89.2%) were married, 94 (84.7%) had obtained a high school degree or below, and 54 (48.6%) were from the countryside. The patients ranked their informational needs (mean: 3.80; SD: 0.91), technical needs (mean: 3.41; SD: 0.85), psychological/emotional needs (mean: 3.15; SD: 1.09), and care coordination and communication needs (mean: 3.12; SD: 0.87) (see supplementary materials 4). The Cronbach’s α coefficient of the total PNSS was 0.93, and all 49 items showed satisfactory item-to-total correlation, with correlation coefficients ranging from 0.43 to 0.84. Cronbach’s α coefficient of the total PNSS-GC was 0.95, and the Guttman Split-half coefficient was 0.81. The top three specific needs were ranked as follows: “protecting my privacy” (81.1%), “telling me the correct way to clean my perineum in daily life” (75.7%) and “reducing the possible discomfort caused by gynecological examination” (71.2%). According to the needs assessment responses by patients, information provision, technical skills guidance about specific symptom management and rebuilding comfort, and maintaining dignity were urgent. The investigation results provided evidence to help identify patients’ perceived common and specific needs, especially the prior needs, to further need management during the perioperative period. This necessary step also provided a certain basis to help construct the application frame and draft, such as what did patients most want to know at a certain stage during the perioperative period and which content could be presented first according to prior needs.

Considering the adaptation and ease of operation of the mobile application interface and on the basis of the above two phases, we formed a 4 first-level, 17 s-level and 47 third-level application index application program draft. The first-level indices were also the source catalog of the application, which contained four different time intervals, named the hospital admission stage, presurgery stage, postsurgery (hospitalization) stage and postsurgery (home interval) stage. The second- and third-level indices corresponded to the subcatalog related to supportive care needs measures under the source catalog of the application. The prototype application (version 1.0) had a mobile phone administration portal for patients and a web-based portal for health care professionals. The mobile phone administration portal contained 5 modules: Quick Assessment, Professional Support, Interactive Platform, Personal Information and Reminders. The web-based portal consisted of Module Management, User Permission Setting and Data Statistics to help manage the mobile phone administration portal. Detailed information on each module is displayed in Table 1.

**Table 1:** Detail functions of modules of the mobile phone portal and web-based portal

Portal	Major modules	Function	Remarks	Timeline
Mobile-based	Quick Assessment	· Assessing the supportive care needs,psychological health at two intervals. · Forming a warning value if existed.	· Measured by the needs and supply part of PNSS, PNSS-GC, and FACT-Cx. · According to the assessment results, tracking warning value record to provide evidence for customized support.	· Respectively on the day of admission, a week after admission.
Mobile-based	Professional Support	· Pushing individual information or services about professional supportive care according to the Quick Assessment and warning value reminders.	· Focus on professional supportive care about informational, technical, psychological/ emotional and care coordination and communication and specific supportive care needs range from hospital admission to post-surgery. · Pop-up messages with quick links of the professional support would display if warning value existed.	· Any time of using the application.
Mobile-based	Interactive Platform	· Pushing specific suggestions and providing a real-time online consultation according to the Quick Assessment and warning value reminders. · Message board and interactive feedback.	· Mainly focus on supportive care needs during home rehabilitation period. · Information records of the interactive platform.	· Per day of using the application.
Mobile-based	Personal Information	· Application login. · Providing basis on information fetch for the backstage.	· Basic login information. · Results of the Quick Assessment. · Notes of the use time, frequency, and support provision.	· On the day of admission.
Mobile-based	Reminders	· Waring value of reminder, the fill-out surveys and messages alert (such as date of the follow-up).	· Pop-up messages launched. · Pushing notification.	· On the day of discharge.
Web-based	User Permission Setting	· Healthcare professional and researcher login port.	· Basic login information.	· On the day of admission.
Web-based	Module Management	· Providing consultation feedback and managing the warning value by healthcare professional.	· Manage the mobile phone administration portal. · Sending message alert. · Waring value management.	· Per day of using the application.
Web-based	Data Statistics	· Primary data of the application influx linked with mobile phone portal.	· Users’ basic login information. · Application time use, frequency and interactive records. · Results of the needs, supply and quality of life.	· A week after using the application.

### Phase 3: application program draft refining

In total, ten experts were invited to revise the structure and content of the prototype. Six experts were engaged in gynecological supportive care provision, while the other four concentrated on research about cancer nursing or women’s psychological behaviors. The experts ranged in age from 30 to 57 years (mean: 42.80; SD: 9.97) and had 8 to 28 years of work experience (mean: 15.60; SD: 9.20). Over half of them had a master’s degree or higher, and the others were chief nurses or chief physicians. A total of 43 recommendations for improvement were subsequently implemented to improve the mobile application interface. The recommendations mainly focused on the index construction, language expression, intervention content and structure of the application (see supplementary material 5). According to the recommendations, we revised and formed 4 first-level, 21 s-level and 42 third-level indices of the application prototype (version 2.0) (the first and second indices are shown in supplementary material 6). Compared with version 1.0, version 2.0 had five second-level indices added and five third-level indices deleted. Part of the interface of WG version 2.0 is shown in Fig. 3.

### Phase 4: application compatibility testing

The prototype application (version 2.0) used Linux of the Android system as the core of the mobile phone console. After completing data interaction between the mobile phone portal and web-based portal, the mobile phone portal for patients would acquire data from the server or save operating data roots from the user interaction to the server. The health care professional portal adopted the web application open-source Spring plus springMVC plus Hibernate as the framework. Then, the mobile system was layered through springMVC. The management of the health care professional portal was displayed through browser access to realize the intuitive display of the system. After testing the compatibility and stability in 5 different mobile phone systems, there was no flashback frequency.

### Phase 5: pilot quasi-experimental testing & users’ additional comments

A total of 14 patients with cervical cancer who were undergoing surgery (both IG and CG were seven, paired with age) and three health care professionals (two gynecological nurses and a gynecological doctor) were invited to participate in the pilot testing. Seven participants in the IG and three health care professionals were asked to download and install the application on the day of patient admission. The mean age of the patients was 48.21 years (SD: 7.19), and the average mobile phone use time was 3.16 (SD: 1.58). Approximately 71.43% of patients were from urban areas and had obtained a middle school degree or above, 78.57% of them had no religious beliefs, and 92.86% were married. The characteristics of the patients in each group are shown in supplementary material 7. The health care professionals ranged in age from 28 to 36 years, and they had 5 to 13 years of working experience. No system instability phenomenon, such as interface splashing, occurred. A total of 294 page views on the patient portal and 10 platform interactions were captured. There were 91 person-time views of the interactive results on the platform.

Table 2 presents the outcome measures at baseline and over time by group. There were no group differences at baseline. The score of the perceived supportive care supply part of both groups increased by a week, as measured by their perceived care coordination and communication supply scores (IG: 2.72 vs. 4.22; CG: 2.60 vs. 3.34), quality of life (physical well-being subscale [IG: 22.00 vs. 27.43; CG: 18.14 vs. 26.57] and cervix subscale [IG: 28.43 vs. 35.29; CG: 30.71 vs. 27.86]). The intervention group had a higher total QoL score than the control group after one week (IG: 76.12 vs. 93.25). Unfortunately, although we had set the warning value to help provide personalized supportive care, no warning value appeared in our pilot study. Overall, the feedback in the user’s additional comments showed that the users had a positive experience with the application, which was designed to be user-friendly and to provide support for postsurgical recovery. The users made suggestions, voicing their expectation about extending the functions of the application and prolonging its usage, as shown in Table 3.

**Table 2:** Score of outcome measures at baseline and over time by group

Variables	Intervention group mean(SD)		Control group mean(SD)	
	Baseline	A week	Baseline	A week
Total score of the PNSS- Needs part	3.30(0.84)	3.88(0.79)	3.08(0.73)	4.07(0.60)
Informational needs	3.85(0.62)	4.31(0.69)	3.56(0.76)	4.30(0.58)
Technical needs	3.34(1.13)	3.82(0.88)	2.95(0.88)	4.10(0.68)
Psychological/ emotional needs	3.09(1.02)	3.93(0.96)	3.09(0.86)	3.79(0.73)
Care coordination and communication needs	3.07(1.04)	3.48(1.09)	2.89(0.86)	4.11(0.82)
Total score of the PNSS-GC- Needs part	4.11(0.47)	3.99(0.59)	3.51(0.81)	3.79(0.78)
Total score of the PNSS- Supply part	2.72(1.00)	4.22(0.46)	2.60(0.50)	3.34(1.07)
Informational needs	2.94(1.02)	4.59(0.25)	3.27(0.66)	3.82(0.92)
Technical needs	2.71(1.16)	4.36(0.32)	2.53(0.34)	3.47(1.09)
Psychological/ emotional needs	2.41(1.39)	3.90(0.55)	2.12(1.07)	3.23(1.35)
Care coordination and communication needs	2.55(1.30)	4.07(0.46)	2.51(0.28)	2.88(1.16)
Total score of the PNSS-GC- Supply part	2.94(1.21)	3.96(0.68)	2.79(0.79)	3.94(0.84)
Total score of the FACT-Cx	76.12(5.85)	93.25(6.33)	76.05(5.79)	86.89(14.16)
Physical well-being	22.00(13.00)^a^	27.43(0.29)^a^	15.00(6.00)^a^	26.71(0.57)^a^
Functional well-being	14.00(6.43)	18.71(3.15)	20.29(5.56)	22.00(2.71)
Social/family well-being	17.57(6.45)	19.29(2.56)	13.71(5.09)	14.14(6.26)
Emotional well-being	16.00(13.00)^a^	20.00(3.00)^a^	11.00(8.00)^a^	18.00(4.00)^a^
Cervix subscale	28.43(4.12)	35.29(2.21)	30.71(3.90)	27.86(8.55)

Note: IG: intervention group, CG: control group. SD: standard deviation. PNSS: professional nursing support scale, PNSS-GC: professional nursing support scale- gynecological cancer, FACT-Cx: functional assessment of cancer therapy- cervix subscale. a: for the non-normal distribution of the data, median (interquartile range) of the two domains of the FACT-Cx was presented

**Table 3:** Themes of users’ experience for the application

Themes	Examples
Positive experience to use the application for patients	“The pelvic floor muscle exercise is very difficult for me to adhere. I do not exercise now. I hope this software can help me for this deficiency…It’s rich in content.” (Patient 1, short for P1)
	“I can also consult doctors if I have problems at home from this software (application). This (application) is achievable. Also, can I communicate with other peers in similar situation as me?…Can I only download this software(application) from the department where I am hospitalized? Would it disappear when I arrive home or not?” (P2)
	“The content of the application is quite rich, for it is hard to judge whether the information I checked on the Internet is true or false. But this (application) is convenient for me to use it directly.” (P3)
	“I feel the content I read is all I need, which is very helpful to me, thank you very much.” (P4)
	“It’s pretty easy to handle, so I’m just going to click in different catalog.” (P5)
	“The interaction from healthcare professionals and peers who had the same disease as me encouraged me a lot. The design module of Interactive Platform is very important and useful.” (P6)
	“The software is free of charge, and the content also make me trust. I am very satisfied.”(P7)
Expectation about function extension concerning the application for patients	“Diet guidance about postoperative, especially staying at home, should be more detailed.” (P1)
	“I am now more than 7 months after surgery. Is it possible that I can continuously use it? Could you please put more content suitable for me at home?” (P2)
	“I tried to ask the doctor a question, but the reply was not very timely. Maybe they were busy with their work. It would be better to reply as soon as possible.” (P3)
	“The first time I logged in, I had to input my height and some other information. It is a little tedious.” (P4)
	“I want to contact patient peers here, but I can’t add friends on this application. If you could add the function to add friends like WeChat, it would be much more convenient.” (P5)
	“I discharged from hospital with a catheter, and I tried to do perineal care with the guidance of the application. But I accidentally pulled so hard I felt like it would be pulled out. I couldn’t figure out what to do next.” (P6)
	“I forgot to see every few days, especially the exercise of pelvic floor muscle, you sent a message to remind me, I had just remembered. It can increase the punch and reward function, so maybe I can hold the mobile phone to exercise every day”. (P7)
Positive experience to use the application for patients for managers	“It’s effectively for screening patients needs in time and an useful pathway to understand some sensitive issues that they won’t ask openly.” (Nurse 2)
	“It’s a good way to help patients actively instead of passively self-report. We trusted each other more.” (The doctor)
	“I can clearly see the needs of patients with the help of the engineer… There were fewer psychosocial needs but more about symptom management.” (Nurse 1)
Expectation about function extension concerning the application for managers	“I can’t always respond to patients’ questions online, and more responded at a certain time. More problems needed to be solved with the help of doctors…Recruiting more doctors and replying in staggered time may help to solve the interaction problem.” (Nurse 2)
	“Patients were mostly consulted about symptom-related problems. Can we consider setting the warning value of symptoms?” (The doctor)

## Discussion

The present study documented the process and feasibility test of a mobile phone application conducted using multiple methods focused on cervical cancer patients’ professional supportive care needs. Before a standard randomized confirmatory trial (RCT) was undertaken, components in the intervention also needed to be identified [18]. This kind of component identification process could compensate for the defects of traditional intervention development approaches, which involved the cycle of constructing an intervention (by evaluating in a standard RCT and performing post hoc analyses to explain how and why it worked) and then constructing a second intervention revision (by evaluating in a new RCT) [18]. Collins et al.’s review of the shortcomings of the traditional approach to intervention development recognized that the cycle of the development process might lead to a delayed effect of the intervention, and an alternative approach of building, optimizing and evaluating eHealth interventions was needed [26].

Here, we used a needs assessment-centered design to identify the intervention components. During the process of literature review, quantitative professional supportive care needs assessment, suggestions on the content and structure of the intervention from expert reviews, the preinstallation of the mobile system prototype, pilot feasibility testing and preliminary exploration of the use experience of users, we presented the cocreation process of an application for patients with cervical cancer and health care professionals. We developed not only a mobile phone for patients but also a web-based administration portal for health care professionals to manage the application. The close cooperation with patients and health care professionals provided valuable insight into critical requirements for both the development and implementation of mobile applications [27].

The formative process of our mobile application had the following advantages. Regarding content design, the application specifically focused on supportive care needs for patients with cervical cancer who were undergoing surgery. On the basis of our previous literature review, 13 of 16 (81%) applications were mainly developed for symptom management, negative emotion management and QoL improvement related to various cancer types, and neither cervical cancer inpatients nor supportive care needs were addressed. Regarding the platform management and function settings, the application was designed to promote online communication or interaction between health care professionals and patients. The alert function and warning value setting were the main innovations of the application, which was expected to help dynamically monitor supportive care needs, provide personalized supportive care services and help self-management. Despite the confirmed effectiveness of previous eHealth application programs, most of the programs were centered around the stage of cervical cancer screening. Only one application developed by Weaver et al. had set the function of sending an alert to the medical portal, and hence, health care professionals could contact the patient offline to provide solutions [28]. On the phase of pilot testing of the application, although we cannot judge the short-term effects of the outcome measures for the small sample, it inspired our morale that we were getting closer to the answer of “Yes, we can!” of a feasibility study [19]. We anticipate various advantages from using an eHealth application, including increased supportive care supply, higher quality of life, and improved supportive care needs in our further trail (Trail registration: Chinese Clinical Trails Registry number ChiCTR, approval number: 2,000,033,316).

### Limitations

There were some limitations in this study. In the literature review phase, only Chinese and English articles were included. This may have introduced language bias, as articles written in other languages with relevant outcomes might have been missed. In the needs-identification phase, the participants were from only two affiliated hospitals. Therefore, the representativeness should be examined with caution. In the expert review phase, only expertise in gynecology and gynecological cancer research was exploited. Consequently, there might have been a lack of authority in obstetrics, although we had collected related recommendations about fertility. In the pilot study phase, the health outcomes needed continuous observation and larger sample size which might add the quasi-experimental effect and statistic analysis power, so that we could only anticipate that the possible effects would indicate in future research. Regrettably, we had not tracked any warning values during the quasi-experimental study, and the limited sample size and short intervention time might be the explanation. Furthermore, we observed the long-term effect of the intervention by increasing the frequency of the intervention. Since the health care professionals flexibly scheduled the online interaction at their convenience, there might have been a delay in response, and the untimely interaction may also have affected the user experience and problem-solving ability. Unfortunately, we did not use any well-known questionnaire to evaluate the user experience, which might have led to relatively subjective evaluation results. We will apply the user experience questionnaire to evaluate the perceived usefulness, perceived ease of use, and user acceptance of the Womb Guard in the evaluation of standard effectiveness. In addition, our program is currently only suitable for patients who have an Android mobile phone system. Future research will continue to expand the application development to different mobile application systems.

## Conclusion

The WG was specifically developed to manage the supportive care needs of patients with cervical cancer who were undergoing surgery. It contained a mobile portal for patients and a web-based administration portal for health care professionals. The application was expected to allow users to identify supportive care needs in the perioperative period, set and track goals for supportive care, and receive professional and evidence-informed health care tips. Our feasibility study seemed to observe the score change of in groups of the outcome measures. The short- and long-term effects of using an eHealth application to increase supportive care supply and improve supportive care needs and quality of life will be investigated in our future trail.

### Electronic supplementary material

Below is the link to the electronic supplementary material.

Supplementary Material 1

## Data Availability

Neither the information in this manuscript nor the analyzed results in this manuscript has been reported previously. SYX is the contact person whom could be contacted for data availability.
