# Supplementary material for: Development of a supportive care needs eHealth application for patients with cervical cancer undergoing surgery: a feasibility study
Source: BMC Health Serv Res. 2024 Jan 2;24:3. doi: 10.1186/s12913-023-10437-3 (PMC10763441; doi:10.1186/s12913-023-10437-3)
Supplement: Supplementary file 1 — Supplementary Material 1 [file 12913_2023_10437_MOESM1_ESM.doc]

**SUPPLEMENTARY INFORMATION**

**Supplementary Material 1**: [Patients’ eligible criteria in phase 1, 2 and 5].

**Supplementary Material 2:** [PRISMA flow diagram of the literature review in phase 1]

**Supplementary Material 3**: [Literature review of application construction in phase 1].

**Supplementary Material 4**: [Characteristics of patients and their needs score in phase 2].

**Supplementary Material 5**: [Overview of expert recommendations in phase 3].

**Supplementary Material 6:** [The first and second indexes of the application in phase 3].

**Supplementary Material 7**: [Characteristics of patients in two groups in phase 5].

**Supplementary information** **1.** Eligible criteria in phase 1, 2 and 5.

| Phase | Inclusion criteria | Exclusion criteria |
| --- | --- | --- |
| Phase 1: Application Program Components Screening | 1. study participants were adults aging 18 and above with a confirmed diagnosis of cancer. 2. studies included the construction methods of the application in detail, and evaluated the effectiveness of the supportive care interventions on mobile phones. | (a) using short messaging service, phone call of smart phones as intervention methods.  (b) only abstracts without access to the full text.  (c) repeatedly published or unpublished grey studies. |
| Phase 2: New Application Program Draft Forming | (a) histopathologically diagnosed with cervical cancer according to the guidelines for the diagnosis and treatment of gynecological malignant tumors formulated by the International Federation of Gynecology and Obstetrics in 2015. | (a) serious physical diseases or mental or cognitive disorders.  (b) Karnofsky functional status score less than 60. |
|  | (b) received or prepared to receive cervical conization, extrafascial total hysterectomy, subextensive total hysterectomy plus pelvic lymphadenectomy, extensive total hysterectomy plus pelvic lymphadenectomy, and/ or para-aortic lymphadenectomy. |  |
|  | (c) aged between 18 and 85 years.  (d) awareness of their cancer diagnosis.  (e) ability to communicate and understand and agree to participate the study. |  |
| Phase 5: Pilot Quasi-experimental Testing & User Experience Collection | (a) histopathologically diagnosed with cervical cancer according to the guidelines for the diagnosis and treatment of gynecological malignant tumors formulated by the International Federation of Gynecology and Obstetrics in 2015. | (a) serious physical diseases or mental or cognitive disorders.  (b) Karnofsky functional status score less than 60. |
|  | (b) prepared to receive cervical conization, extrafascial total hysterectomy, subextensive total hysterectomy plus pelvic lymphadenectomy, extensive total hysterectomy plus pelvic lymphadenectomy, and/ or para-aortic lymphadenectomy. |  |
|  | (c) aged between 18 and 85 years.  (d) awareness of their cancer diagnosis.  (e) ability to communicate and understand and agree to participate the study.  (f) used the Android smartphone daily (over 2 hours per day). |  |

**
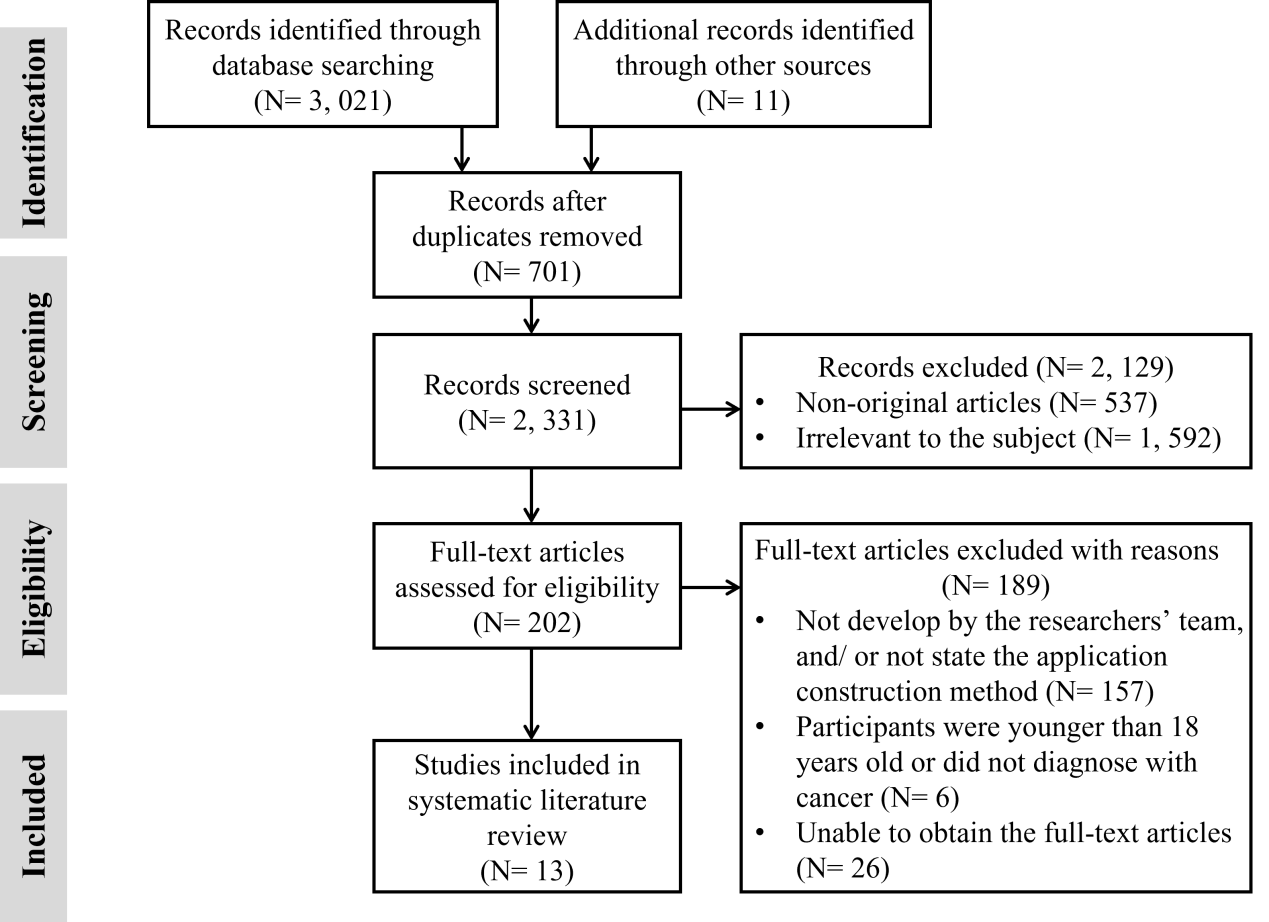
**

**Supplementary information 2.** PRISMA flow diagram of the literature review.

**Supplementary information 3.**

**3.1 Search Strategy**

Total nine databases (four Chinese and five English databases) were searched. The search was restricted to orginal articles targeting cancer patients related to supportive care and mHealth application usability evaluation. The final search was conducted from January 2000 to December 2019. A two-step search strategy was used. The initial step included a search using the keywords and Medical Subject Headings terms. The search terms included population (neoplasms, cancer, carcinoma), mHealth intervention (application, health application, telehealth, telemedicine, mobile application, mobile applications, mobile application, m-health, mhealth, e-health, mobile health, smartphone application, smartphone applications). This was to ensure that all relevant studies were not missed. The index terms and keywords of a specific concept were combined using the Boolean operator “OR”, while the differing concepts were then searched together using the Boolean operator “AND”. Hand search for unpublished studies from China National Knowledge Internet and ProQuest for graduate research. The reference lists of the eligible studies and relevant systematic reviews of the topic were hand searched to identify additional studies for screening.

**3.2 Literature review results of application construction in cancer patients.**

| **Author (Year, Country), cancer type** | | **Application name** | **Application development methods** | **Study methods** | **Application modules/ topics** | **QualSyst**  **score** |
| --- | --- | --- | --- | --- | --- | --- |
| Duman-Lubberding,  et al (2016, Netherlands). Head and neck cancer | OncoKompas | | 1. Semi-structured interviews from the view of health care professionals and cancer survivors. 2. Application developed together with mixed teams (cancer survivors, medical specialists and allied health professionals). 3. In-depth interview for collecting using experience. | A pre-post test and 2 weeks later in-depth interview.  ·Sample size: (pretest: n=106 vs posttest: n=53)  · Sampling: invited by an oncology nurse or head and neck surgeon.  ·Time point: 2 weeks  ·Attrition rate: 36%  ·Outcomes:  Primary- application usage and satisfaction  Secondary- clinical variables and health-related quality of life | ·Psychological quality of life  ·Physical quality of life  ·Social quality of life  ·Healthy lifestyle  ·Life questions  ·Head and neck cancer | Qualitative quality evaluation, 0.80 |
| Fu, et al (2016, USA). Breast cancer | The-Optimal-Lymph-Flow health IT system - TOLF | | 1. Needs assessment of patients and experts. 2. Developing the application prototype. 3. A pre-post test for users and semi-structured interview for collecting using experience. | Randomized controlled trail  ·Sample size: (intervention: n=53 vs control: n=51)  ·Sampling: computer generated block randomization with variable block sizes.  ·Time point: 3 months  ·Attrition rate: 13.5%  ·Outcomes:  Primary- utilization of eHealth services  Secondary- confidence (self-efficacy). | ·Muscle contraction exercise  ·Shoulder exercise  ·Large muscle group exercise  ·Nutrition  ·Sleep, etc. | Quantitative quality evaluation, 0.86 |
| Gustavell T, et al (2019, Sweden). Pancreatic cancer | Interaktor | | 1. Symptom assessment. 2. Application development of the prototype on the basis of risk assessment model. 3. A feasibility evaluation and in-depth interview for collecting using experience. | A feasibility study and 4 weeks later in-depth interview.  ·Sample size: 6  ·Sampling: screening from a scheduled to undergo pancreaticoduodenectomy at a university hospital during a period of 18 weeks.  ·Time point: 4 weeks  ·Attrition rate: NA  ·Outcomes: symptom and alarts, application usage and satisfaction | ·Regular assessment of self-reported symptoms and problems  ·A connection to a monitoring Web interface.  ·A risk assessment model for alerts on frequent or distressing symptoms.  ·Continuous access to evidence-based self-care advice and links to relevant Web sites.  ·Graphs for the patients to view a history of their symptom reporting. | Qualitative quality evaluation, 0.90 |
| Han et al (2017, China). Breast cancer | Rehabilitation of upper limb function- Recovery assistant | | 1. Application development of the prototype on the basis of the existing “Rehabilitation Assistant” application and patients’ needs assessment. 2. A pre-post test for patients and nurse evaluation. | A pre-post test.  ·Sample size: 46  ·Sampling: selected according to the admission date.  ·Time point: 6 months  ·Attrition rate: 0%  ·Outcomes:  Primary- exercise compliance, upper extremities edema incidence  Secondary- patients satisfaction for nursing care | ·Automatic Reminders  ·Health Tips  ·Content to be done  ·Rehabilitation guidance  ·Question-and-answer follow-up  ·Recovery record  ·Health alliance | Quantitative quality evaluation, 0.86 |
| Harder et al (2017, UK). Breast cancer | bWell | | 1. Focus groups with patients to identify user needs. 2. Literature review. 3. Preliminary survery testing to obtain user feedback. | A user-centred approach and co-creation process of bWell.  ·Sample size: 9  ·Sampling: not described  ·Time point: 8 weeks  ·Attrition rate: NA  ·Outcomes: user needs and requirements for bWell | ·Information provision  ·Video demonstrations  ·Graded tasks  ·Record exercises performed  ·Motivational statements  ·Push notifications, including opt-in opt-out option  ·Behaviour and progress tracking  ·Diary function  ·Section FAQs  ·Web links | Qualitative quality evaluation, 0.90 |
| Kearney, et al (2008, UK). Lung, breast or colorectal cancer | Application for symptom management for patients receiving outpatient chemotherapy-ASyMS© | | 1. Literature review 2. Expert consultation 3. Patients’ perceptions of pre-participation. | Randomized controlled trail and usability test.  ·Sample size: n=112  ·Sampling: an automated interactive voice response telephone randomisation system  ·Time point: baseline, chemotherapy  pre-cycle 2, pre-cycle 3, pre-cycle 4 and pre-cycle 5  ·Attrition rate: (intervention:14.8%-46.3% vs control: 14.3%-48.2%)  ·Outcomes: chemotherapy-related symptoms | ·Symptom assessment  ·Information storage  ·Feedback from health professionals  ·Health education  ·Amber alert | Qualitative quality evaluation, 0.90 |
| Klasnja, et al (2016, USA). Breast cancer | HealthWeaver | | 1. Extensive fieldwork 2. Application development of the prototype 3. Design group discussion | Design group discussion.  ·Sample size: n=5  ·Sampling: not described  ·Time point: 3 weeks  ·Attrition rate: NA  ·Outcomes: experiences with application use | ·Daily check-ins to track well-being and symptoms  ·Calendar events  ·Logs to monitor medications, pain, and surgery drains  ·Notes for quick capture of care related information. | Qualitative quality evaluation, 0.90 |
| Ruland, et al (2013, USA). Breast and prostate cancer | WebChoice | | 1.Application interface design on the basis of literature review and clinical practice guidelines.  2. Qualitative interviews for users.  3. Randomized controlled trail and usability test | 1. Expert nurses discussion Forum; patients forum for group discussion and application use (n=10).  2. Randomized controlled trail and usability test  ·Sample size: n=103  ·Sampling: not described  ·Time point: 6 months  ·Attrition rate: NA  ·Outcomes: patterns of use & Perceived usefulness | ·Assessment component  ·Self-management component  ·Information component  ·Communication component  ·Diary | Quantitative quality evaluation, 0.86 |
| Sundberg et al (2015, Sweden). Prostate cancer | ICT- platform | | 1. Literature review 2. Interviews with patients and health care professionals | An usability test and focus-group discussion.  ·Sample size: n=10  ·Sampling: convenience sample  ·Time point: 6 weeks  ·Attrition rate: 10%  ·Outcomes: symptom questionnaire and the self-care advice relevant and ease of use and acceptability of the application | ·Access to relevant information  ·Hard to know which websites to rely on  ·Alarm made it easy to handle problems  ·Contacted by nurse  ·Alert | Qualitative quality evaluation, 1.00/ Quantitative quality evaluation, 0.86 |
| Timmerman, et al (2016, Netherlands). Lung cancer | Information and Communication Technology (ICT) | | 1. Semi-structured interviews for patients and healthcare professionals | Semi-structured interviews and the usability evaluation  (7 lung cancer patients and 10 healthcare professionals). | ·Information on disease and treatment  ·Information on lifestyle  ·Ambulant monitoring  ·Exercise  ·Contact with fellow patients  ·Contact with healthcare professionals  ·Medication prescription and reminders  ·Others | Qualitative quality evaluation, 0.90/ Quantitative quality evaluation, 0.86 |
| Vogel, et al  (2019, Australia). Ovarian cancer | Mobile Application for Genetic Information on Cancer (mAGIC) | | 1. Application development of the prototype on the basisi of the Fogg Behavior Model and the Health Belief Model.   1. Focus groups to identify the preferences and familiarity of the application. | Randomized controlled trail  ·Sample size: (intervention: n=53 vs control: n=51)  ·Sampling: computer generated block randomization with variable block sizes  ·Time point: 3 months  ·Attrition rate: 13.5%  ·Outcomes:  Primary- utilization of eHealth services  Secondary- confidence (self-efficacy) | ·Genetic counseling  ·Genetic testing  ·Cancer genetics & personal health  ·Cancer genetics & my family  ·Taking care of yourself  ·Preparing for a genetic counseling applicationointment  ·Summary | Quantitative quality evaluation, 0.86 |
| Weaver, et al (2014, UK) Breast or colorectal cancer | Datacap | | 1. Literature review 2. A pilot, one arm, single-centre clinical study | A pilot, one arm, single-centre clinical study  ·Sample size: 26  ·Sampling: NA  ·Time point: 3 months  ·Attrition rate: 13.5%  ·Outcomes:  Primary- utilization of eHealth services  Secondary- confidence (self-efficacy) | ·Symptom record  ·Diet record  ·Dose record  ·Nurse-patient interaction | Quantitative quality evaluation, 0.86 |
| Zhu, et al (2018, China). Breast cancer | Bcs | | 1. Literature review 2. Application development of the prototype on the basisi of Bandura’s self-efficacy and social exchange theory. 3. Semi-structured interview for collecting using experience. | Qualitative interview  ·Sample size: 13  ·Sampling: purposive sampling.  ·Time point: 12 weeks  ·Attrition rate: 13.5%  ·Outcomes: utilization of application and self-efficacy | ·Learning forum  ·Discussion forum  ·Ask-the-Expert forum  ·Personal Stories forum | Qualitative quality evaluation, 0.80 |

Note: NA: not applicable.

**3.3 List of the included articles in phase 1.**

1. Duman-Lubberding S, van Uden-Kraan CF, Jansen F, Witte BI, van der Velden LA, Lacko M, Cuijpers P, Leemans CR, Verdonck-de Leeuw IM. Feasibility of an eHealth application “OncoKompas” to improve personalized survivorship cancer care. *Supportive Care in Cancer*, 2016, 24(5): 2163-2171. http://doi:10.1007/s00520-015-3004-2
2. Fu M R, Axelrod D, Guth AA, Rampertaap K, El-Shammaa N, Hiotis K, Scagliola J, Yu G, Wang Y. MHealth Self-care Interventions: Managing Symptoms Following Breast Cancer Treatment. *Mhealth*, 2016, 2: 28. http://doi:10.21037/mhealth.2016.07.03
3. [Gustavell T](http://apps.webofknowledge.com/OutboundService.do?SID=8CdEEhClVIySeAYh4Ya&mode=rrcAuthorRecordService&action=go&product=WOS&daisIds=15501343), [Langius-Eklof A](http://apps.webofknowledge.com/OutboundService.do?SID=8CdEEhClVIySeAYh4Ya&mode=rrcAuthorRecordService&action=go&product=WOS&daisIds=807247), [Wengstrom Y](http://apps.webofknowledge.com/OutboundService.do?SID=8CdEEhClVIySeAYh4Ya&mode=rrcAuthorRecordService&action=go&product=WOS&daisIds=280984), Segersvärd R, Sundberg K. Development and feasibility of an interactive smartphone APP for early assessment and management of symptoms following pancreaticoduodenectomy. *Cancer Nursing*, 2019,42(3):e1-e10. http://doi:10.1097/NCC.0000000000000584
4. Han N, Liu Y, Li Y, Li X, Wang Y, Song H. Effect of the application on improving postoperative upper extremity function rehabilitation inpatients with breast cancer. *Chinese Journal of Nursing*, 2017, 52(3): 267-270. http://doi:10.3761/j.issn.0254-1769.2017.03.002
5. Harder H, Holroyd P, Burkinshaw L, Watten P, Zammit C, Harris PR, Good A, Jenkins V. User-centred approach to developing bWell, a mobile APP for arm and shoulder exercises after breast cancer treatment. *Journal of Cancer Survivorship*, 2017,11(6): 732-742. http://doi:10.1007/s11764-017-0630-3
6. Kearney N, Mccann L, Norrie J, Taylor L, Gray P, McGee-Lennon M, Sage M, Miller M, Maguire R. Evaluation of a mobile phone based, advanced symptom management system (ASyMS) in the management of chemotherapy related toxicity. *Supportive Care in Cancer*, 2009, 17(4): 437-444. http://doi:10.1007/s00520-008-0515-0
7. Klasnja P, Hartzler A, Powell C, Phan G, Pratt W. Health Weaver Mobile: Designing a mobile tool for managing personal health information during cancer care. *AMIA Annual Symposium Proceedings*, 2010,2010:392-396.
8. Ruland C, Maffei R, Borosund E, Børøsund E, Krahn A, Andersen T, Grimsbø GH. Evaluation of different features of an eHealth application for personalized illness management support cancer patients’ use and appraisal of usefulness. *International Journal Of Medical Informatics*, 2013, 82(7): 593-603. http://doi:10.1016/j.ijmedinf.2013.02.007
9. Sundberg K, Eklöf AL, Blomberg K, Isaksson AK, Wengström Y. Feasibility of an interactive ICT-platform for early assessment and management of patient-reported symptoms during radiotherapy for prostate cancer. *European Journal Of Oncology Nursing*, 2015, 19(5): 523-528. http://doi:10.1016/j.ejon.2015.02.013
10. Timmerman J G, Tönis TM, Dekker-van Weering MG, Stuiver MM, Wouters MWJM, van Harten WH, Hermens HJ, Vollenbroek-Hutten MMR. Co-creation of an ICT-supported cancer rehabilitation application for resected lung cancer survivors: design and evaluation. *BMC Health Service Research*, 2016, 16: 155. http://doi:10.1186/s12913-016-1385-7
11. Vogel R, Niendorf K, Petzel S, Lee H, Teoh D, Blaes AH, Argenta P, Rivard C, Winterhoff B, Lee HY, Geller MA. A patient-centered mobile health application to motivate use of genetic counseling among women with ovarian cancer: a pilot randomized controlled trial. *Gynecologic Oncology,* 2019, 153(1): 100-107. http://doi:10.1016/j.ygyno.2019.01.019
12. Weaver A, Love SB, Larsen M, M Shanyinde, Waters R, Grainger L, Shearwood V, Brooks C, Gibson O, Young AM, Tarassenko L. A pilot study: dose adaptation of capecitabine using mobile phone toxicity monitoring-supporting patients in their homes. *Supportive Care in Cancer*, 2014, 22(10): 2677-2685. http://doi:10.1007/s00520-014-2224-1
13. Zhu J, Ebert L, Guo D, Yang S, Han Q, Chan SWC. Mobile breast cancer e-Support program for Chinese women with breast cancer undergoing chemotherapy (Part 1): qualitative study of women's perceptions. *JMIR Mhealth and Uhealth*, 2018,6(4):E85. http://doi:10.2196/mhealth.9311

Supplementary information 4. Characteristics of patients with cervical cancer and the needs score. (N=111)

| Variables | Statistic |
| --- | --- |
| Age (year) a | 47.94 (8.10) |
| Place of residence  City  County  Town  Rural area | 27 (24.32%)  13(11.71%)  17(15.32%)  54(48.65%) |
| Education level  Primary school or below  Junior middle school  High middle school  College or above | 49(44.14%)  45(40.54%)  13(11.71%)  4(3.61%) |
| Marriage  Unmarried  Married  Widowed or divorced | 0(0.00%)  99(89.19%)  12(10.81%) |
| Family monthly income (RMB)  Over 5000   1. 4999 2. 2999 3. 999   Less than 499 | 18(16.22%)  52(46.85%)  31(27.92%)  4(3.61%)  6(5.40%) |
| Pausimenia  Yes  No | 76(68.47%)  35(31.53%) |
| Disease stage  I  II  III  IV | 76(68.47%)  30(27.02%)  4(3.61%)  1(0.90%) |
| Informational needs a | 3.92 (1.17) |
| Technical needs b | 3.41 (0.85) |
| Psychological/ emotional needs a | 3.38 (1.85) |
| Care coordination and communication needs b | 3.12 (0.87) |
| Total score of the PNSS b | 3.36 (0.79) |
| Total score of the PNSS-GC b | 3.18 (1.04) |

Note: a Continuous variables are presented as the median and interquartile range (IQR) for non-normality distribution. b Continuous variables are presented as the men and standard deviation for normality distribution. The rest variables were categorized variables.

Supplementary information 5. Overview of expert recommendations of the application draft.

| Aspects | Expert recommendations | Reasons and remarks |
| --- | --- | --- |
| Index construction | · (N= 9) Adding information about presurgery and forming 4 first-level indexes. | · Because of the prolonged stay in the hospital before surgery, the intervention of patients during this period could optimize the nurse-patient relationship, and patients would be more familiar with the use of the mobile application. |
| Language expression | · (N= 10) Replacing the expression “cervical cancer” or “cancer” with “disease”.  · (N= 6) Video exercise training such as functional exercise of pelvic floor muscles could be displayed. | · For ethical and cultural consideration.  · For the consideration of improving exercise compliance. |
| Intervention content added | · (N= 7) Details of “dietary precautions” after surgery.  · (N= 4) Information about sexual issue guidance and self-management of catheters after surgery. | · Detailed dietary recipes could be listed for patient options.  · Information about sensitive issues could release the possible anxiety to some extent. |
| Structure of the application | · (N= 7) Possibility of providing outpatient scheduling information and realizing the function of online registration. | · Because of the failed connection of the information interface of hospitals, the function of online registration could not be realized presently. The gynecological clinic schedule and official WeChat of a cooperating hospital were provided as a remedy. |

**Supplementary information 6.** The first and second-level indexes of the application.

| The first-level indexes | The second-level indexes |
| --- | --- |
| Hospital admission | · Admission notice (informational needs)  · Introduction of the medical team members (care coordination and communication needs)  · Cancer related information (informational needs)  · Recommended examination (technical needs) |
| Presurgery | · Surgery introduction (informational needs)  · [Preoperative preparation](javascript:;) (technical needs)  · Cancer specific needs (specific supportive care needs)  · Emotional relaxation Techniques (psychological/ emotional needs) |
| Post-surgery (hospitalization) | · Coping with symptoms after surgery (technical needs)  · Information about postoperative care (informational needs)  · Discharge guidance (informational needs)  · Emotional relaxation Techniques (psychological/ emotional needs)  · Cancer specific needs (specific supportive care needs) |
| Post-surgery (home interval) | · Coping with symptoms at home (technical needs)  · Diet and exercise guidance (informational needs)  · Disease prevention (informational needs)  · Other therapy method (informational needs)  · Appointments and follow-up visits (care coordination and communication needs)  · Introduction of supporting groups or institutions (care coordination and communication needs)  · Emotional relaxation Techniques (psychological/ emotional needs)  · Cancer specific needs (specific supportive care needs) |

Supplementary information 7. Characteristics of patients in phase 5. (N=14)

| Variables | Statistics | |
| --- | --- | --- |
|  | Intervention group (N=7) | Control group (N=7) |
| Age (year) (mean (SD)) | 48.43 (7.44) | 48.00 (7.53) |
| Place of residence  City  County  Town  Rural area | 2(28.57%)  0(0.00%)  3(42.86%)  2(28.57%) | 4(57.14%)  1(14.28%)  0(0.00%)  2 (28.57%) |
| Education level  Primary school or below  Junior middle school  High middle school  College or above | 3(42.86%)  3(42.86%)  1(14.29%)  0(0.00%) | 1(14.29%)  4(57.14%)  2 (28.57%)  0(0.00%) |
| Marriage  Unmarried  Married  Widowed or divorced | 0(0.00%)  7(100.00%)  0(0.00%) | 0(0.00%)  6(85.7%)  1(14.29%) |
| Family monthly income (RMB)  Over 5000   1. 4999 2. 2999 3. 999   Less than 499 | 1(14.28%)  2(28.57%)  3(42.86%)  1(14.29%)  0(0.00%) | 2(28.57%)  1(14.28%)  3(42.86%)  0(0.00%)  1(14.29%) |
| Disease stage  I  II | 4(57.14%)  3(42.86%) | 5(71.43%)  2(28.57%) |
| Time spent for using the application per day (hour) (mean (SD)) | 3.40 (1.98) | 2.93(1.17) |
